# Improvement of hyperlipidemia by aerobic exercise in mice through a regulatory effect of miR-21a-5p on its target genes

**DOI:** 10.1038/s41598-021-91583-8

**Published:** 2021-06-07

**Authors:** Jinfeng Zhao, Yicun Song, Yu Zeng, Longchang Chen, Feng Yan, Anping Chen, Baoai Wu, Yaxin Wang

**Affiliations:** 1 School of Physical Education, Shanxi University, Taiyuan, Shanxi China; 2 Department of Exercise Physiology, Beijing Sports University, Beijing, China

**Keywords:** Molecular biology, Physiology

## Abstract

Hyperlipidemia is a risk factor for cardiovascular disease, and miR-21a-5p plays an important role in the occurrence and progression of hyperlipidemia. Here, we aimed to investigate the mechanism of aerobic exercise improved hyperlipidemia through enhancing miR-21a-5p expression. In this study, high-fat/high-cholesterol diet mice received 8 weeks of aerobic exercise intervention, then we collected plasma and liver samples, we found that there had a notable improvement in weight gain, blood lipid level, and liver steatosis in hyperlipidemia mice after 8 weeks of aerobic exercise intervention. Besides, aerobic exercise significantly up-regulated the expression of miR-21a-5p and provoked favorable changes in the expression of target genes. Knockdown of miR-21a-5p resulted in dysregulation of lipid metabolism and increased expression of FABP7, HMGCR, ACAT1, and OLR1. While aerobic exercise could alleviate miR-21a-5p knock-down induced lipid metabolism disorder. Taken together, these results demonstrated that aerobic exercise improved hyperlipidemia through miR-21a-5p-induced inhibition of target genes FABP7, HMGCR, ACAT1, and OLR1.

## Introduction

Hyperlipidemia is characterized by elevated levels of plasma triglycerides and cholesterol. However, disorders of lipid metabolism are risk factors for cardiovascular diseases such as atherosclerosis, stroke, and coronary heart disease^1^.

A growing body of evidence suggests that non-coding RNAs, such as Long non-coding RNAs (lnc-RNAs), microRNAs (miRs), contribute to the development of hyperlipidemia. MiRs as important targets for regulating lipid metabolism and homeostasis have been a hot topic in recent years^2^. MiRs are small non-coding RNAs consisting of 20–22 nucleotides (nt) that interact with short motifs in the 3' untranslated region (UTR) of target genes, causing translational repression and/or mRNA destabilization^3^. MiR-21a is one of the first mammalian microRNAs identified, which involves many physiological processes and relates to a variety of diseases, one of its most representative roles is to regulate lipid metabolism^4^. Many types of research have demonstrated that the expression of miR-21a-5p was downregulated in nonalcoholic fatty liver patients or high-fat-fed mice and miR-21a-5p knock-out can lead to hepatic steatosis, which accelerated atherosclerosis, plaque necrosis, and vascular inflammation. Thus, miR-21a-5p is a potential target for lipid metabolism regulation^5^.

MiR-21a-5p affects lipid metabolism mainly by regulating the expression of genes related to lipid homeostasis. A single miRNA has multiple targets and simultaneously regulates target mRNAs involved in a physiological pathway^6^. In many studies, we have found that the target genes of miR-21a-5p involved in lipid metabolism, including fatty acid-binding protein 7 (FABP7)^7^, 3-hydroxy-3-methylglutaryl-coenzyme A reductase (HMGCR)^5^, peroxisome proliferator-activated receptor α (PPARα)^8^ and phosphatase and tensin homolog deleted on chromosome 10 (PTEN)^9^. Also, bio-informatic prediction using TargetScan, miRTarBase revealed acetyl-CoA acetyltransferase 1 (ACAT1) and oxidized low-density lipoprotein receptor 1 (OLR1) are potential targets of miR-21a-5p and closely relate to cholesterol metabolism.

As a non-pharmaceutical intervention, aerobic exercise can effectively prevent obesity, improve diabetes and cardiovascular disease, and many studies have demonstrated that aerobic exercise can reduce the pathogenesis and progression of hyperlipidemia through different processes. Maxi Meissner et al. suggested that voluntary wheel running ameliorates cholesterol metabolism, mainly by promoting its conversion into bile acids or decreasing intestinal cholesterol absorption^10^. Other researches showed that aerobic exercise can inhibit cholesterol synthesis by reducing the expression and activity of HMGCR^11^. In addition, our previous studies showed that aerobic exercise reduced the expression of PCSK9, an important gene for cholesterol metabolism, and enhanced LDLR expression^12^. Now some studies confirm that exercise could exert its beneficial effects in the improvement of lipid metabolism by the regulation of miRNA biology^13,14^, but the underlying mechanism of miRNA in mediating the protective effect of exercise against lipid accumulation remains largely unclear.

In this study, we focused on the role of miR-21a-5p in regulating lipid metabolism by affecting its target genes FABP7, PPARα, PTEN, HMGCR, ACAT1, and OLR1. And we want to know whether aerobic exercise can affect the expression of miR-21a-5p, to change the expression of its target genes, and achieve the goal of lowering lipid accumulation.

## Materials and methods

### Animal study

**Figure 1:**
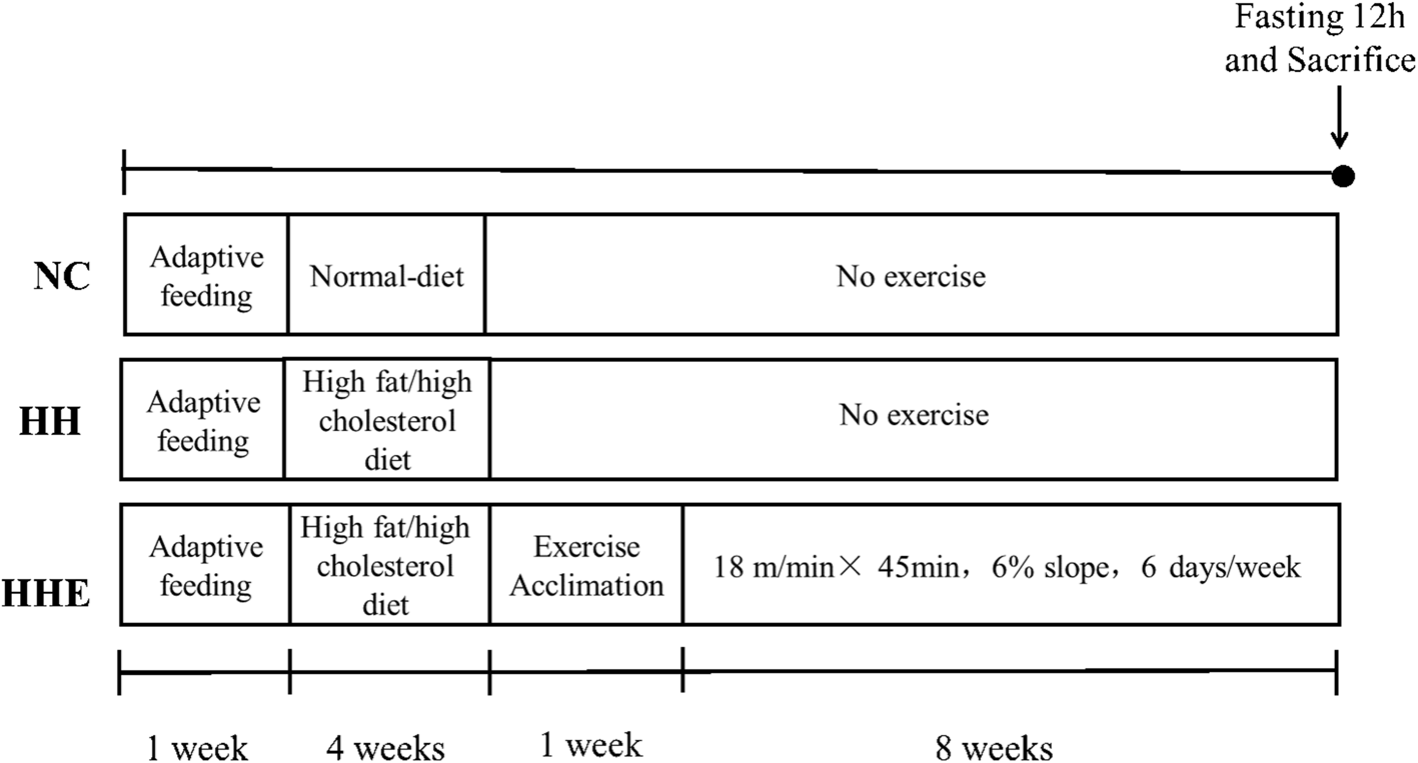
Design of the experiment.

SPF 8-week-old male C57BL/6J mice (n = 30) were provided by Beijing vital river laboratory animal technology biotech (Beijing, China) and fed in China institute for radiation protection (CIRP), the animal license number was SCXK (Beijing) 2016-0006, weighing 21.4 ± 0.92 g. After one week of adaptive feeding, the mice were randomly divided into 3 groups, 10 in each group. Mice in the normal control group (NC) were fed a chow diet, the high-fat/high cholesterol diet group (HH) were fed a high-fat/high-cholesterol diet, and the high-fat/high cholesterol diet plus aerobic exercise group (HHE) received aerobic exercise intervention for 8 weeks based on a high-fat/ high-cholesterol diet (Fig. 1). Raising condition: the temperature was 20–26 °C, the humidity was 40–60%, light and dark cycle every 12 h, all mice had free access to tap water and food. After the final exercise, the mice fasted overnight and were then sacrificed. Blood samples were collected in tubes containing EDTA and centrifuged at 3500 rpm at 4 °C for 15 min, and the plasma was transferred to new tubes, stored at − 80 °C. Mice livers were quickly removed, snap-frozen in liquid nitrogen after washed with cold phosphate-buffered saline (PBS), stored at − 80 °C for future analysis.

Anti-miR-21a-5p adeno-associated virus vector (AAV9) driven by TBG promoter (100 μL, 1.95 × 10^12^ vg/ml) (Sangon Biotech, Shanghai, China) was injected into the tail vein to specifically inhibit the expression of miR-21a-5p in the liver (n = 10), and an empty vector was served as negative control (n = 10). Five weeks after injection, miR-21a-5p KD mice on a chow diet or high-fat diet were given a 5 weeks aerobic exercise intervention, then mice were sacrificed after the last exercise. All animal studies were conducted following the Ethics Committee of Scientific Research in Shanxi University, experiments conformed to local and international guidelines on the ethical use of animals, and this study adhered to the ARRIVE guidelines.

### Exercise program

After 4 weeks of high fat/high cholesterol feeding, HHE groups underwent a 1-week treadmill exercise training, followed by 8 weeks of formal aerobic exercise. The intensity of aerobic exercise was determined to be 18 m/min × 45 min, 6% slope, 6 days/week, and each exercise had a 5-min warm-up, a total of 8 weeks.

### Lipid Profile

Serum total cholesterol (TC), triglyceride (TG), LDL cholesterol (LDL-C), HDL cholesterol (HDL-C) was assayed by spectrophotometer (UV-6100 s, Mapada, Shanghai, China) according to the kit’s instructions (Nanjing Jian cheng biotech, China). The content of TG and TC were measured at 510 nm, LDL-C and HDL-C were measured at 546 nm.

### Histologic analysis

Liver tissues were fixed in 10% neutral buffered formalin, then sections were stained with standard hematoxylin–eosin (H&E), the degree of liver steatosis was observed under a microscope.

### Real-time quantitative RT-PCR

Total RNA was isolated from mice liver samples using a spin column animal total RNA purification kit (Sangon Biotech, Shanghai, China) under the manufacturer's instructions. M-MuLV first-strand cDNA synthesis kit was used for reverse transcription into cDNA with total RNA as a template. MiRNA was isolated with a miRNeasy Mini kit and reverse transcription was performed using the Tail-added reverse transcription kit (Sangon Biotech, Shanghai, China). Quantitative PCR was performed on a LightCycler480 system (Roche, Switzerland) using TB Green *premix Ex Taq* II mix (TaKaRa, Dalian, China), gene-specific primers were listed in Table 1, β-actin and U6 small nuclear RNA (snRNA) was used as an internal control.

**Table 1:** Gene-specific primers.

Gene	Direction	Sequence (5'–3')
FABP7	Forward	GAAACCAGCATAGATGACAGAA
	Reverse	TAACAGCGAACAGCAACGATA
PPARα	Forward	TACTGCCGTTTTCACAAGTGC
	Reverse	AGGTCGTGTTCACAGGTAAGA
HMGCR	Forward	TCTGGCAGTCAGTGGGAACTATT
	Reverse	CCTCGTCCTTCGATCCAATTT
PTEN	Forward	TGGATTCGACTTAGACTTGACCT
	Reverse	GCGGTGTCATAATGTCTCTCAG
ACAT1	Forward	CAGGAAGTAAGATGCCTGGAAC
	Reverse	TTCACCCCCTTGGATGACATT
OLR1	Forward	CAAGATGAAGCCTGCGAATGA
	Reverse	ACCTGGCGTAATTGTGTCCAC
Dicer1	Forward	GGTCCTTTCTTTGGACTGCCA
	Reverse	GCGATGAACGTCTTCCCTGA
miR-21a-5p	Forward	CCGCGTAGCTTATCAGACTGATGTTGA
miR-33a	Forward	GTGCATTGTAGTTGCATTGCA
miR-34	Forward	AATCAGCAAGTATACTGCCCT
miR-29a	Forward	TAGCACCATCTGAAATCGGTTA
miR-27	Forward	AGTTCACAGTGGCTAAGTTCCGC
miR-200c	Forward	CCGTAATACTGCCGGGTAATGATGGA
miR-122	Forward	TGGAGTGTGACAATGGTGTTTG

### Western blot

Mice liver tissues were lysed in RIPA lysis buffer, the total soluble protein was quantified using the BCA Protein Assay kit (Beyotime, Shanghai, China). Proteins (40 μg) were separated by sodium dodecyl sulfate–polyacrylamide gel electrophoresis (SDS-PAGE), then cropped and transferred to PVDF membranes, blocked for 1 h with 5% nonfat milk. After overnight incubation with primary antibody (1:1000 anti-FABP7, anti-HMGCR, anti-PTEN, anti-PPARα, anti-ACAT1, anti-OLR1) (Proteintech Group Inc, Wuhan, China), membranes were washed and incubated with HRP-conjugated secondary antibody (1:5000) (Boster Biotech, Wuhan, China). Then the signals were detected using an ECL detection kit (Applygen Technologies Inc, Beijing, China) and imaged in a chemiluminescence-measuring instrument (ChemiDoc XRS + , Bio-Rad, USA).

### Statistical analysis

All the experimental data were presented as mean ± standard error (SD). Comparisons were performed with one-*way*, *two*-*way* ANOVA or Student’s t-test. The analysis program used SPSS (25.0). P-values were considered significant at < 0.05.

## Results

### Aerobic exercise beneficially affect body weight and plasma lipoprotein profile

**Figure 2:**
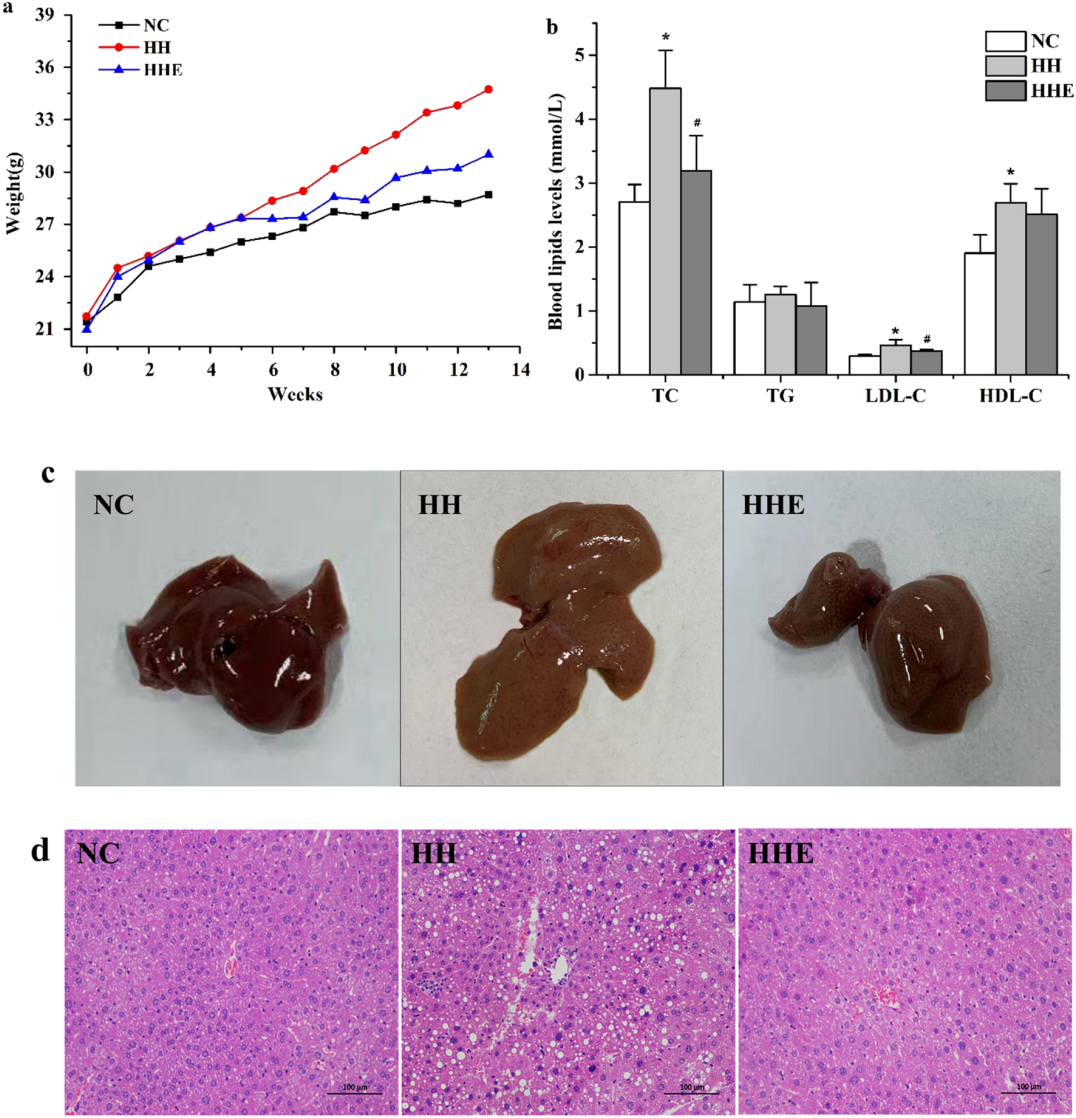
Protective effect of aerobic exercise on lipid metabolism disorders. (**a**) The trend of body weight change of mice in each group. (**b**) Serum lipids of mice in each group. (**c**) Visual observation of mice liver tissue. (**d**) HE staining to observe liver steatosis grade. *P < 0.05 VS NC group, # P < 0.05 VS HH group.

A high-fat/ high-cholesterol diet-induced hyperlipidemia mouse model was used to evaluate the lipid-lowering effects of aerobic exercise. As can be clearly seen from Fig. 2a, the weight of HH group mice showed a rapidly rising trend, significantly higher than that of the NC group at the end of the experiment(P < 0.05). However, from 6 to 13 weeks of aerobic exercise intervention, the bodyweight of the HHE group was significantly diminished than HH group. Consistently, the liver weight of the HH group was also significantly higher and decreased significantly after exercise, but there was no significant difference in the liver-to-body weight ratio (Fig. S1). Besides, after 12 weeks of a high-fat /high-cholesterol diet, fasting plasma lipids were significantly increased in HH mice than NC group, representing prominent serum lipids accumulation. While the serum lipid levels of HH group mice recovered significantly when underwent the aerobic exercise intervention for 8 weeks, indicated that aerobic exercise provoked favorable changes in plasma lipid level (Fig. 2b).

### Aerobic exercise improved steatosis of high fat/high cholesterol diet mice

The livers of the NC group were bright red with smooth surface and sharp edges by visual inspection, while the livers of the HH group were yellowish-brown with dull edges, we can make a preliminary judgment that the livers were covered with lipids after the high-fat/high cholesterol diet (Fig. 2c). Histologic analysis by H&E staining displayed that the liver of hyperlipidemia mice revealed severe histopathological alterations described as numerous small cytoplasmic vacuoles associated with punctate necrosis of liver cells and infiltration of inflammatory cells. In contrast, we found that after aerobic exercise intervention, the number of liver lipid droplets were decreased and the size became smaller, the arrangement of liver cells was neat. (Fig. 2d).

### Aerobic exercise beneficially regulated the expression miR-21a-5p and its target genes

**Figure 3:**
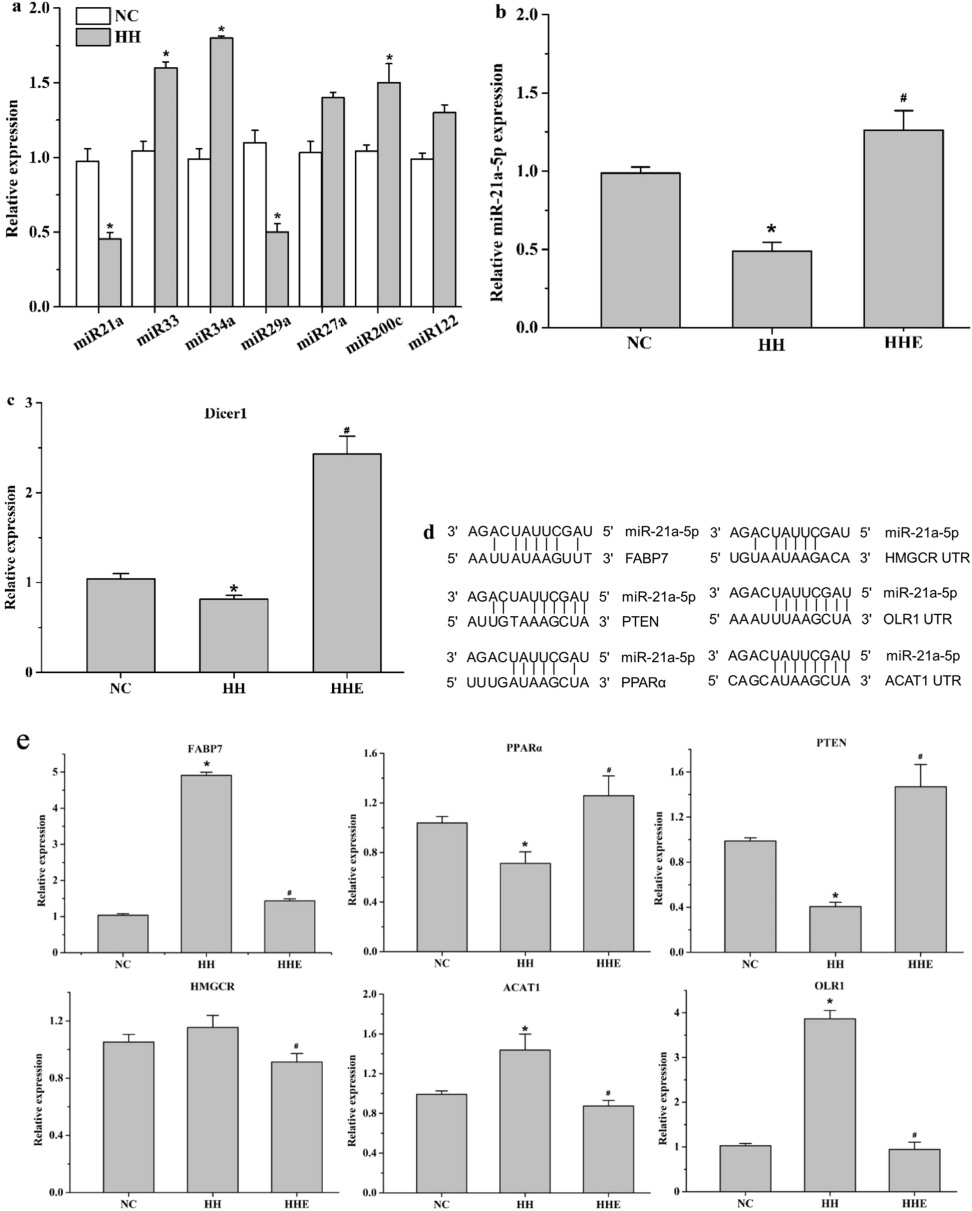

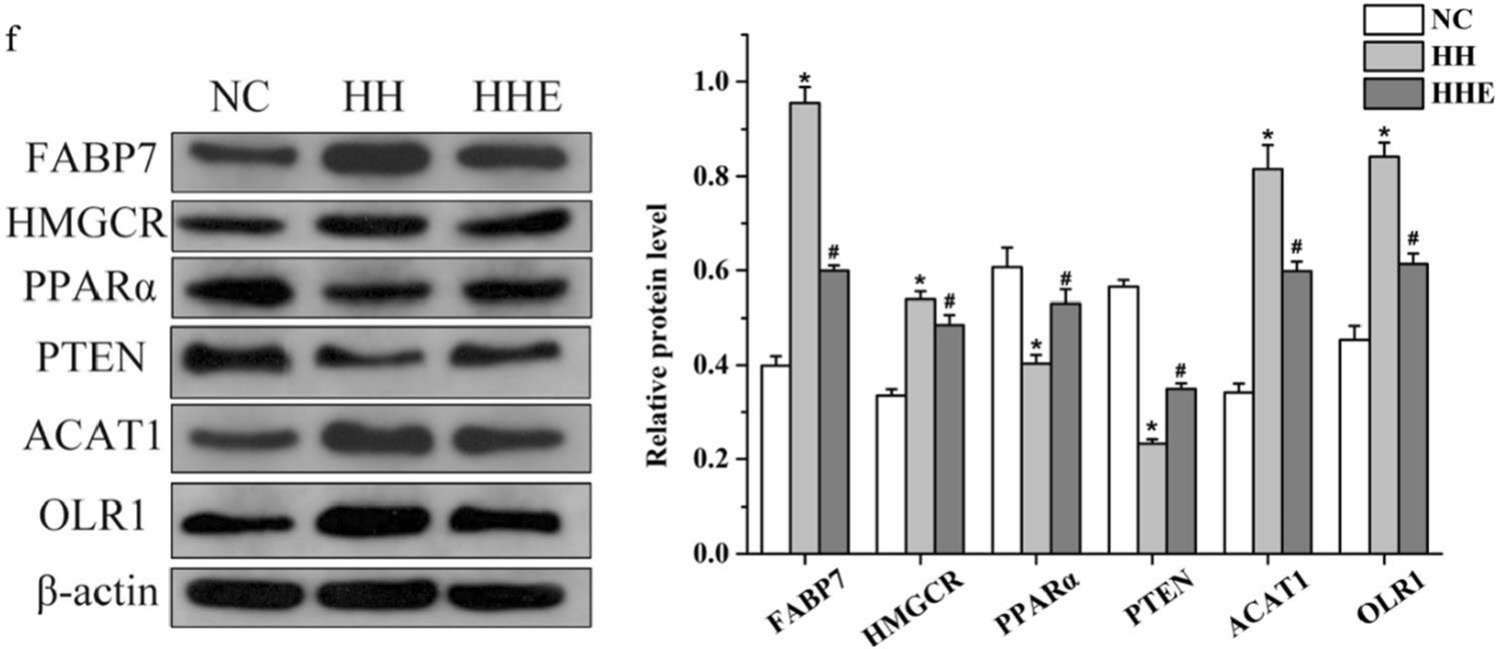
Effects of aerobic exercise on the expression of miR-21a-5p and its target genes. (**a**) Relative levels of miRNAs in the liver of mice fed a high-fat diet. (**b**) qPCR was used to detect the expression of miR-21a-5p in NC, HH, and HHE groups. (**c**) The mRNA levels of Dicer1. (**d**) Schematic diagram of miR-21a-5p seed sequence with the 3′-UTR binding site of target genes. (**e**) The mRNA levels of miR-21a-5p targets were detected by QPCR. (**f**) The protein levels of miR-21a-5p targets were determined by western blot, original western blot gel figures are presented in Supplementary data. *P < 0.05 VS NC group, # P < 0.05 VS HH group.

Numerous miRNAs have been reported to involve in lipid regulation. In our previous experiment, we have detected the expression of miR-33, miR-34a, miR-21a, miR-29a, miR-27a, miR-200c, miR-122, the results showed that these miRNAs had a significantly different expression in the HH group, and there was a notable diminished of miR-21a-5p (p < 0.01) (Fig. 3a), which was consistent with other studies that miR-21a-5p expression was decreased in patients with NAFLD or mice on a high-fat diet. Whereas, after 8 weeks of aerobic exercise intervention, miR-21a-5p was significantly elevated (p < 0.05) (Fig. 3b). Also, we detected the expression of Dicer1, an enzyme for miRNA processing, was decreased in the HH group, but highly elevated in the HHE group (Fig. 3c).

Previous studies have confirmed that FABP7, HMGCR, PPARα, and PTEN are direct targets of miR-21a-5p. In addition, we predicted other target genes of miR-21a-5p by TargetScan and found that ACAT1 and OLR1 have a putative binding site in its 3′untranslated region (UTR) (Fig. 3d). To determine whether the target genes of miR-21a-5p responded to aerobic exercise, we measured their mRNA levels after aerobic exercise. Dates revealed that the expression of PPARα, PTEN declined in the HH group, but significantly increased after exercise intervention. On the contrary, the mRNA levels of FABP7, HMGCR, ACAT1, and OLR1 elevated significantly in the HH group, but at 8 weeks post-exercise intervention, all downregulated (P < 0.05) (Fig. 3e). And we also detected the protein levels of miR-21a-5p targets, which were consistent with mRNA levels (Fig. 3f).

Among them, FABP7, PPARα, and PTEN are closely related to fat metabolism, and HMGCR, ACAT1, and OLR1 can regulate cholesterol synthesis and metabolism. Thus, we believed that one of the mechanisms that aerobic exercise improves hyperlipidemia maybe by influencing the expression of genes related to lipid metabolism.

### miR-21a-5p regulated the expression of genes involved in lipid metabolism

**Figure 4:**
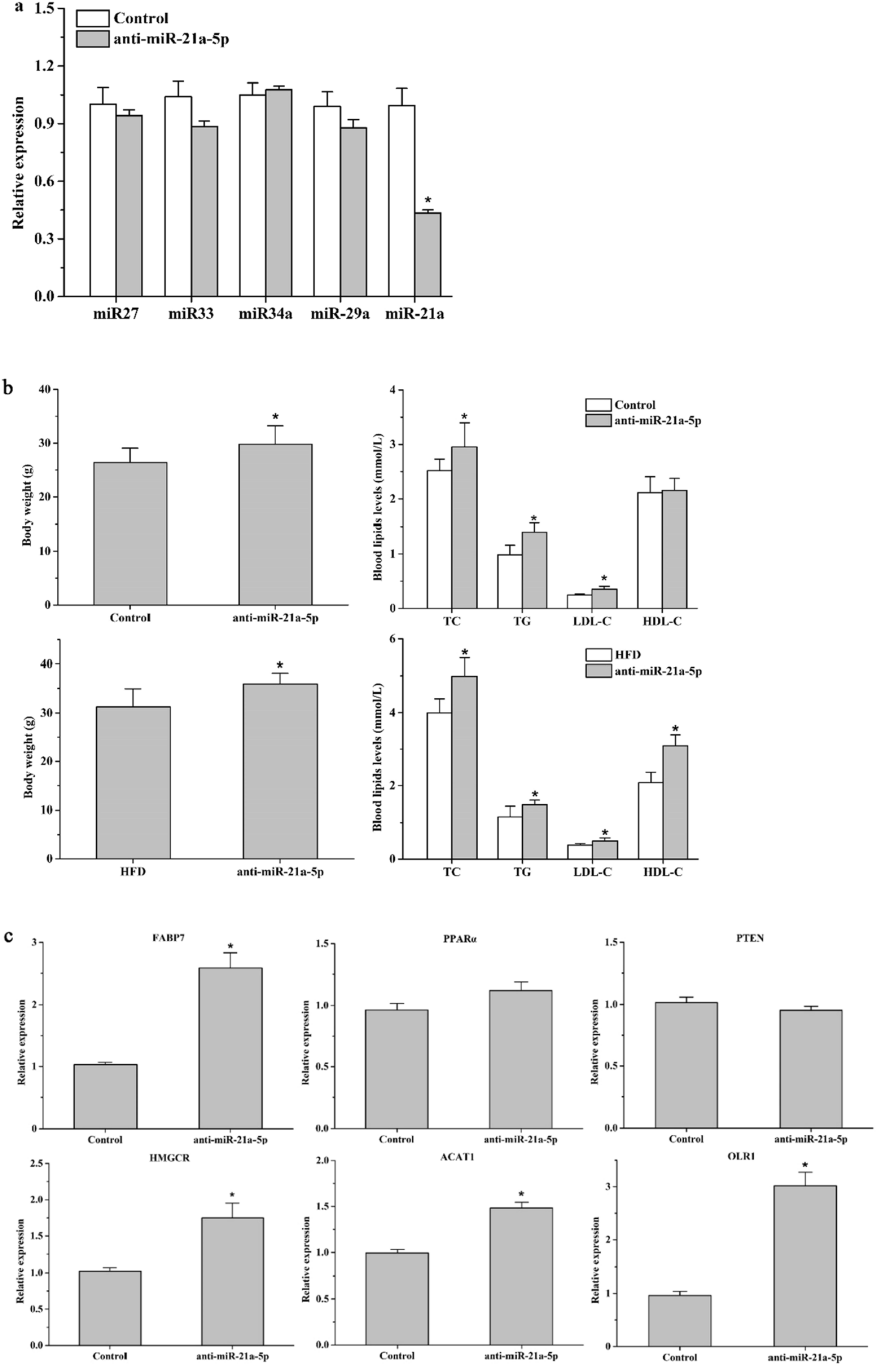
Knock-down of miR-21a-5p led to lipid metabolism disorder. (**a**) The expression of miR-27, miR-34a, miR-33, miR-29a, miR-21a-5p after injected anti-miR-21a-5p AAV 5 weeks. (**b**) The body weight and serum lipid levels of miR-21a-5p KD mice on chow diet or high fat diet. (**c**) mRNA levels of target genes in miR-21a-5p KD mice. *P < 0.05 VS Control or HFD group.

To further examine the impact of miR-21a-5p on hepatic steatosis and measure the effect of miR-21a-5p on its target genes, we specifically inhibited miR-21a-5p in mice liver using an AAV9 encoding anti-miR-21a-5p. In order to detect the specific inhibitory effect, we examined the expression of miR-33, miR-34, miR-27, miR-29a and miR-21a-5p, and found that compared with the control group, miR-27, miR-33, miR-34a miR-29 had no obvious changes, the expression of miR-21a-5p decreased significantly (Fig. 4a).

MiR-21a-5p KD mice on a chow diet or high-fat diet showed increased body weight and serum lipid levels (P < 0.05) (Fig. 4a,b), these results indicated the mechanisms of lipid metabolism in vivo were impaired, resulting in lipid accumulation. Next, we verified the relationship between the expression of FABP7, PPARα, HMGCR, PTEN, ACAT1, OLR1, and miR-21a-5p through quantified the expression of these targets in miR-21a-5p KD mice. We can see that inhibition of miR-21a-5p significantly increased the FABP7, HMGCR, ACAT1, and OLR1 mRNA levels, while PPARα, PTEN had no significant changes (Fig. 4c). Therefore, we believed that miR-21a-5p regulates lipid metabolism mainly by affecting the expression of FABP7, HMGCR, ACAT1, and OLR1.

### Rescue effect of aerobic exercise on miR-21a-5p KD mice

**Figure 5:**
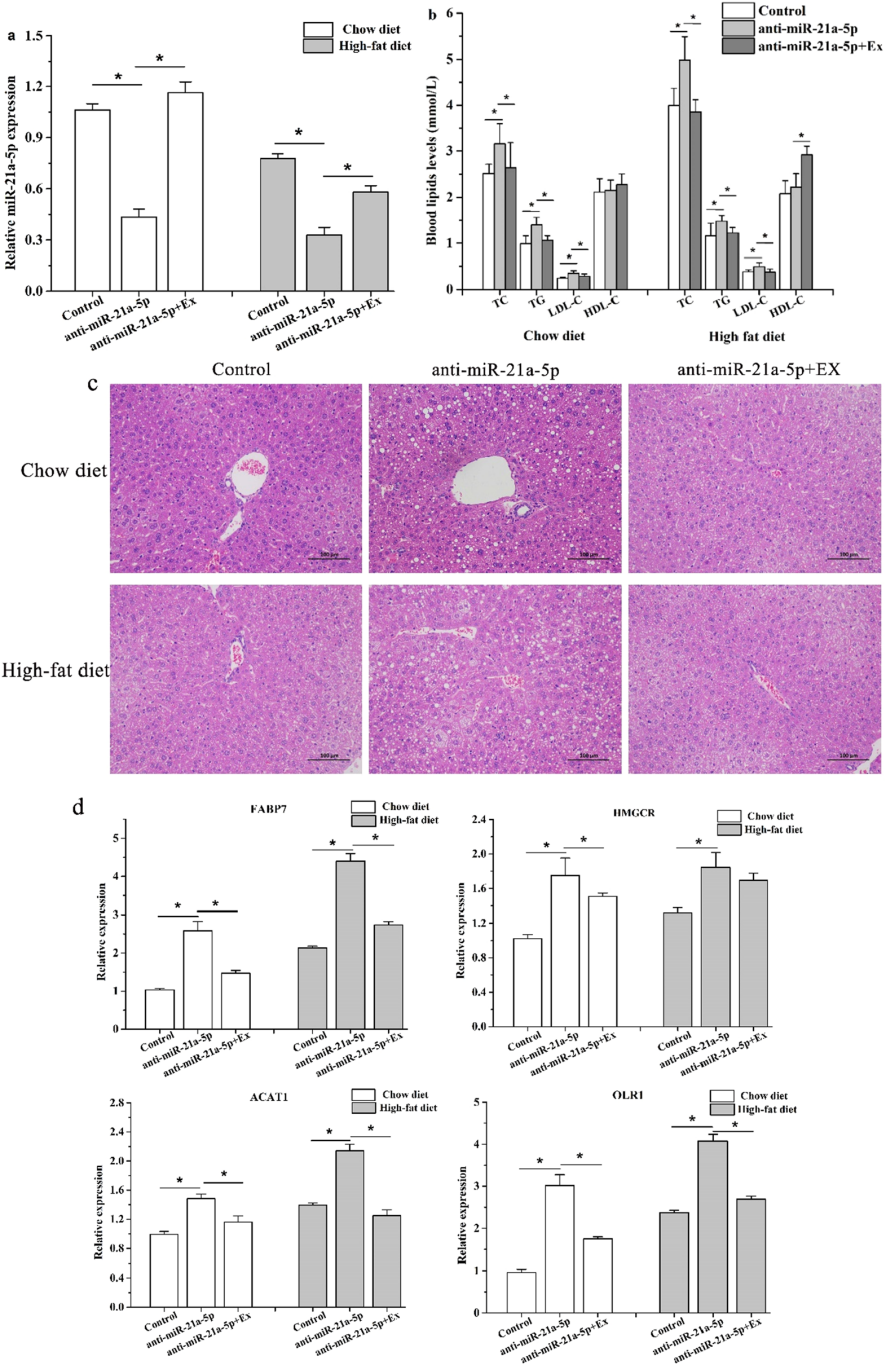
Improvement effects of aerobic exercise on miR-21a-5p KD mice with a chow diet or high-fat diet. (**a**) The expression of miR-21a-5p (**b**) The serum lipid levels (**c**) H&E staining to assess liver steatosis (**d**) The mRNA levels of target genes in miR-21a-5p KD mice after exercise intervention. *P < 0.05 VS Control group, # P < 0.05 VS anti-miR-21a-5p group.

The fact that aerobic exercise can effectively reduce lipid accumulation has been proved, and the relationship between miR-21a-5p and lipid metabolism was well established, hence, it was essential to focus on whether the increased miR-21a-5p levels induced by aerobic exercise affect lipid accumulation, we conducted a five-week aerobic exercise intervention on miR-21a-5p KD mice with chow diet or high-fat diet and found that the expression of miR-21a-5p was regained after aerobic exercise (Fig. 5a). Moreover, we found that the blood lipid level and liver steatosis of miR-21a-5p KD mice were significantly improved (Fig. 5b,c), indicating that aerobic exercise could alleviate the lipid accumulation caused by the loss of miR-21a-5p, these results strongly verified that the involvement of miR-21a-5p in the improvement of hyperlipidemia by aerobic exercise. Additionally, we found that aerobic exercise can reduce the mRNA levels of FABP7, HMGCR, ACAT1, and OLR1 in miR-21KD mice (P < 0.05) (Fig. 5d). Therefore, we showed that one of the mechanisms by which aerobic exercise regulates lipid metabolism was to up-regulate the expression of miR-21a-5p, thereby suppressing the expression of its target genes FABP7, HMGCR, ACAT1, and OLR1.

## Discussion

In our experiment, the weight gain, plasma lipid levels, and liver pathology of high fat/high cholesterol diet mice had a favorable shift after 8 weeks of aerobic exercise. And we think one of the mechanisms by which aerobic exercise improved hyperlipidemia was to up-regulate the expression of miR-21a-5p, thus inhibited the expression of target genes FABP7, HMGCR, ACAT1, and OLR1, which were closely related to lipid metabolism.

MiR-21a-5p plays a key role in suppressing the occurrence and development of hyperlipidemia. In our study, when we specifically suppressed the expression of miR-21a-5p, in which other miRNAs did not show significant changes, we found that the expression of FABP7, HMGCR, ACAT1, and OLR1 was decreased. The expression of multiple genes could be regulated by one microRNA, and the combination of several microRNAs could regulate one gene. FABP7, HMGCR, ACAT1, and OLR1 are all direct targets of miR-21a-5p, of course, it is undeniable that they are also regulated by other miRNAs, like miR-29a. Exercise can upregulate the expression of miR-21a-5p, thereby inhibiting its target gene and exerting a regulatory effect on lipid metabolism. The protective effect of exercise may also act on other miRNAs, which requires further study. Xiao et al. provided compelling evidence suggesting that miR-212 might be a novel therapeutic target mimicking the benefit of exercise in the treatment of NAFLD^14^. Besides, aerobic exercise and statins can induce the expression of miR-146a, thereby reducing the TRAF and TLR4 signaling pathways and vascular inflammatory damage in atherosclerosis, confirming the protective effect of aerobic exercise on vascular diseases^15^. Furthermore, exercise could effectively reduce the tumor volume, lower levels of proinflammatory cytokines TNFα, increase the anti-inflammatory cytokine IL-10 by regulating miR-21a-5p expression^16^. Taken together, these researches indicated that aerobic exercise could play a variety of physiological regulatory roles through miRNA, and miR-21a-5p is an important target for exercise to improve lipid metabolism.

As we all know Dicer1 is essential for miRNA maturation, the disruption of the Dicer1 results in the loss of mature miRNAs. Ming-Xia LIU et al. have shown that the expression of Dicer1 was significantly reduced in the NASH mice model and inversely associated with hepatic FC level. Through further research, they found that serum lipid disorder caused by Dicer1 deletion may be due to the decreased expression of miR-29, which improves nonalcoholic fatty liver disease by suppressing HMGCR expression^17^. This study made us wonder aerobic exercise increased the expression of miR-21a-5p may be through the enhancement of Dicer1 expression. Interestingly, Aaron P Russell et al. have found that after 60 min acute endurance exercise, miRNA processing complex, including Drosha, Dicer, and Exportin-5, were significantly up-regulated by 35, 35 and, 30%, respectively^18^. And our study also showed that Dicer1 expression decreased in the HH group, while increased after exercise intervention, which may be one of the reasons why exercise promoted miR-21a-5p expression.

Researches on the mechanism of miRNAs regulate target genes expression are generally believed that miRNA is to induce post-transcriptional gene silencing through mRNA degradation or translation repression. Mihnea et al. summarized seven unconventional ways in which miRNAs can exert regulatory functions^19^, and recently the function of miRNA has been extended to transcriptional levels, either directly or indirectly^20^. In our study, the expression of FABP7, HMGCR, ACAT1, and OLR1 were increased in miR-21a-5p KD mice, indicated that miR-21a-5p played a negative regulatory role on these target genes, whether this negative regulatory effect is at the post-transcriptional level or the transcriptional level still needs further in-depth study. Importantly, recent studies have indicated that miRNAs have a dual role. When it is located in the cytoplasm, miRNAs inhibit gene expression, when it is in the nucleus, it can activate gene expression by binding to the target gene enhancer^21^. If the miRNA is distributed in both the cytoplasm and the nucleus, then it is likely to perform multiple functions on the target genes. And researches have revealed that miR-21a-5p could be detected both in the cytosol and in the nucleus^22^, so we will pay more attention to how miR-21a-5p regulates the expression of target genes in the nucleus in the future.

There are some limitations to our study, which should be acknowledged. Firstly, our animal procedures were informed by other studies^17^, but one point worth discussing is the duration of fasting on the last day. Many articles on lipid metabolism mention overnight fasting followed by the sacrifice of mice to collect samples for subsequent analysis, but fasting is a key factor affecting energy metabolism, so further studies are needed to determine whether 12 h of fasting at night affects the metabolic capacity of the liver and whether there is an appropriate time to elute the effect of the diet on blood lipids. Then, in our study, it was found that there was no significant change in PTEN and PPARα in miR-21a-5p KD mice, we thought the result might reflect a compensatory mechanism of the organism under the stimulation of pathological stress of dyslipidemia after the miR-21a-5p knockdown. And there may be other pathways were implicated in the regulation of aerobic exercise on the expression of PTEN and PPARα other than miR-21a-5p^23^, this need requires further in-depth study.

In conclusion, this study provided new insights into the mechanism of aerobic exercise regulated lipid metabolism through miRNA. Our work identified miR-21a-5p as a potential regulator of aerobic exercise affecting lipid metabolism, achieved the favorable goal in hyperlipidemia by synergistically inhibiting the expression of target genes FABP7, HMGCR, ACAT1, and OLR1.

## Supplementary Information

Supplementary Information.
