# Supplementary material for: Improvement of hyperlipidemia by aerobic exercise in mice through a regulatory effect of miR-21a-5p on its target genes
Source: Sci Rep. 2021 Jun 7;11:11966. doi: 10.1038/s41598-021-91583-8 (PMC8184843; doi:10.1038/s41598-021-91583-8)
Supplement: Supplementary file 1 — Supplementary Information. [file 41598_2021_91583_MOESM1_ESM.docx]

**Supplementary Date**

**Improvement of** **hyperlipidemia by aerobic exercise in mice through a regulatory effect of miR-21a-5p on its target genes**

Jinfeng Zhao^1^, Yicun Song^1^, Yu Zeng^1^, Longchang Chen^1^, Feng Yan^1^, Anping Chen^1^, Baoai Wu^1,*^, Yaxin Wang^2,*^

^1^ Department of Sports and Education, Shanxi University, Taiyuan, Shanxi, China

^2^ Department of Exercise Physiology, Beijing Sports University, Beijing, China

***** Correspondence: Baoai Wu [469203910@qq.com](mailto:469203910@qq.com); Yaxin Wang 305993851@qq.com

**Short title:** Exercise improves hyperlipidemia by upregulating miR-21a-5p


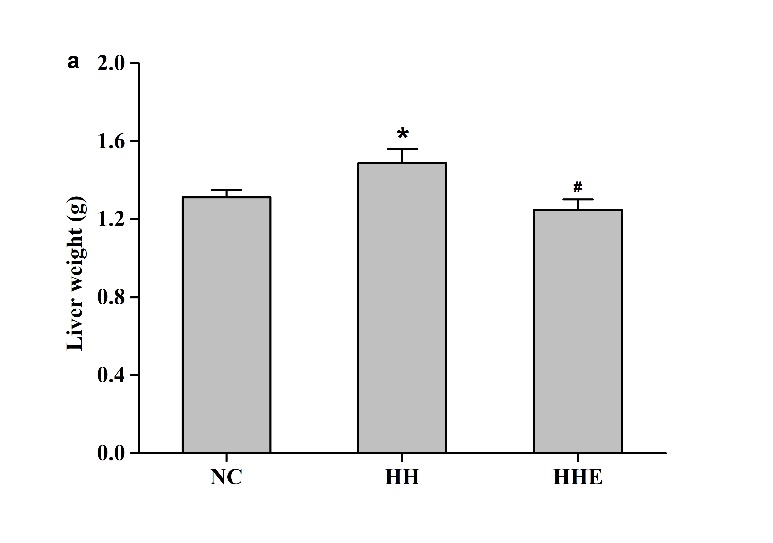

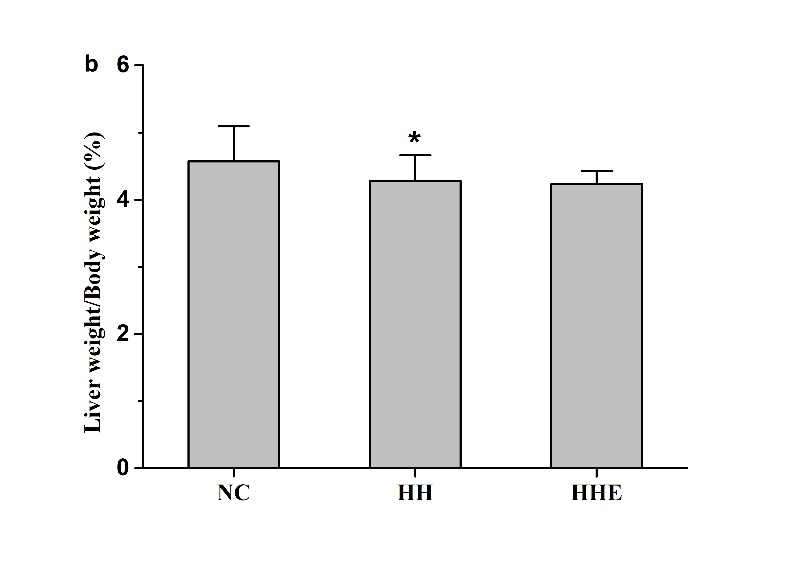


Figure S1 Changes in liver weight after high-fat diet or aerobic intervention. (a) The average weight of livers from mice in different groups. (b) The ratio of liver weight to body weight.


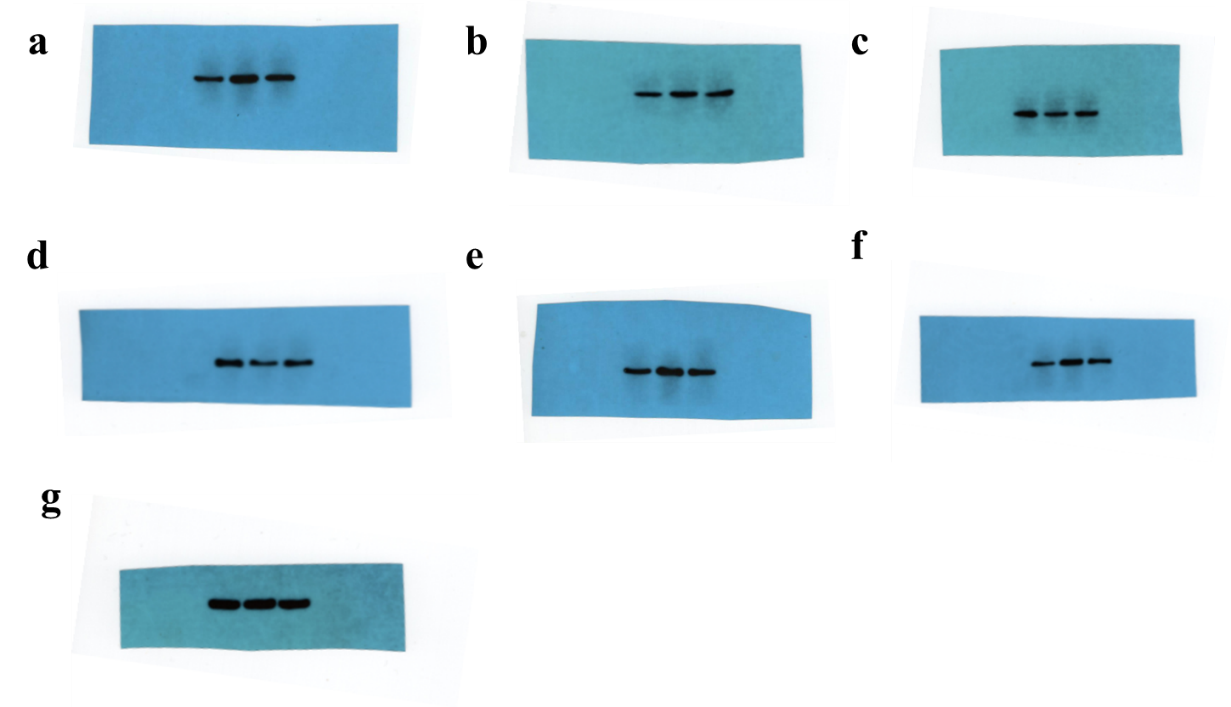


Figure S2. The original gel image after the gel was cropped and transferred to the membrane and imaged after antibody incubation. (A) FABP7, (B) HMGCR, (C) PPARα, (D) PTEN, (E) ACAT1, (F) OLR1.
